# Attention-Deficit/Hyperactivity Disorder and Mortality Risk in Taiwan

**DOI:** 10.1001/jamanetworkopen.2019.8714

**Published:** 2019-08-07

**Authors:** Vincent Chin-Hung Chen, Hsiang-Lin Chan, Shu-I Wu, Meng Lee, Mong-Liang Lu, Hsin-Yi Liang, Michael E. Dewey, Robert Stewart, Charles Tzu-Chi Lee

**Affiliations:** 1 Department of Psychiatry, Health Information and Epidemiology Laboratory, Chang Gung Memorial Hospital at Chiayi, Puzi, Taiwan; 2 Department of Psychiatry, Chang Gung University, Taoyuan, Taiwan; 3 Department of Child Psychiatry, Linkou Chang Gung Memorial Hospital, Taoyuan, Taiwan; 4 Department of Medicine, Mackay Medical College, New Taipei City, Taiwan; 5 Department of Psychiatry, Mackay Memorial Hospital, New Taipei City, Taiwan; 6 Department of Neurology, Chang Gung University and Chang Gung Memorial Hospital at Chiayi, Puzi, Taiwan; 7 Department of Psychiatry, Wan-Fang Hospital, Taipei, Taiwan; 8 School of Medicine, College of Medicine, Taipei Medical University, Taipei, Taiwan; 9 Institute of Psychiatry, Psychology and Neuroscience, King’s College London, London, United Kingdom; 10 South London and Maudsley NHS Foundation Trust, London, United Kingdom; 11 Department of Health Promotion and Health Education, National Taiwan Normal University, Taipei, Taiwan

## Abstract

**Importance:**

Few studies have investigated levels of mortality in patients with attention-deficit/hyperactivity disorder (ADHD), and findings have been inconsistent and lacking information on specific causes of deaths.

**Objective:**

To investigate the association between ADHD and causes of death in Taiwan.

**Design, Setting, and Participants:**

A nationwide population-based cohort study was conducted using a cross-national Taiwanese registry. The ADHD group comprised 275 980 individuals aged 4 to 44 years with a new diagnosis between January 1, 2000, and December 31, 2012. All individuals with ADHD were compared with 1 931 860 sex- and age-matched controls without ADHD.

**Exposures:**

The association between ADHD and mortality was analyzed using a Cox regression model that controlled for sex, age, residence, insurance premium, outpatient visits, congenital anomaly, intellectual disability, depression disorder, autism, substance use disorder, conduct disorder, and oppositional defiant disorder. The analysis of suicide, unintentional injury, homicide, and natural-cause mortality was performed by a competing risk adjusted Cox regression controlling for other causes of mortality and potential confounding factors.

**Main Outcomes and Measures:**

Data on mortality from all causes, suicide, unintentional injury, homicide, and natural causes collected from a national mortality database.

**Results:**

There were 275 980 individuals with ADHD and 1 931 860 comparison individuals without ADHD in this study. Sex and age at index date were matched. The mean (SD) age was 9.61 (5.74) years for both groups. Most of the participants were male (209 406 in the ADHD group; 1 465 842 in the non-ADHD group; 75.88% for both groups). A total of 4321 participants from both cohorts died during the follow-up period (15.1 million person-years), including 727 (0.26%) from the ADHD group and 3594 (0.19%) from the non-ADHD group. Of those who died, 546 (75.1%) in the ADHD group and 2852 (79.4%) in the non-ADHD group were male. After adjusting for potential confounders, compared with the non-ADHD group, patients with ADHD showed higher overall mortality (adjusted hazard ratio, 1.07; 95% CI, 1.00-1.17) and higher injury-cause mortality from suicide (adjusted hazard ratio, 2.09; 95% CI, 1.62-2.71), unintentional injury (adjusted hazard ratio, 1.30; 95% CI, 1.10-1.52), and homicide (adjusted hazard ratio, 2.00; 95% CI, 1.09-3.68). No increased risk of natural-cause mortality was observed after adjustment.

**Conclusions and Relevance:**

In this study, ADHD was associated with higher injury-cause mortality, particularly that due to suicide, unintentional injury, and homicide. Although the risk of injury mortality was significantly higher in patients with ADHD than in the non-ADHD group, the absolute risk of mortality was low.

## Key Points

**Question:**

Is attention-deficit/hyperactivity disorder (ADHD) associated with higher early mortality risk among patients in Taiwan?

**Findings:**

In this Taiwan nationwide population-based cohort study, there were 275 980 patients with ADHD and 1 931 860 matched controls. After adjustment for potential confounders, patients with ADHD had significantly elevated early mortality risk for suicide, homicide, and unintentional injuries compared with the non-ADHD group.

**Meaning:**

These findings suggest that ADHD may be associated with excess mortality from injury causes.

## Teaser

This nationwide cohort study in Taiwan evaluates whether attention-deficit/hyperactivity disorder (ADHD) is associated with higher risk of mortality from all causes, suicide, unintentional injury, homicide, and natural causes.

## Introduction

Attention-deficit/hyperactivity disorder (ADHD) is a common neurodevelopmental disorder with an estimated worldwide prevalence of 7.2% among children and adolescents.^1^ In 60% of individuals, symptoms of ADHD persist into adulthood.^2^ Attention-deficit/hyperactivity disorder has been found to be associated with a range of adverse outcomes, including worse academic achievement, other mental disorders, substance use disorders, criminality, unemployment, and increased health system costs and use.^3^ Associations between ADHD and adverse physical health–related outcomes,^4^ such as diabetes^5^ and traumatic brain injury,^6^ have also been described.

Despite functional impairments and adverse health consequences associated with ADHD, investigations of mortality associations are scant, although all suggest higher risk.^7,8,9^ For example, Barbaresi et al^7^ reported a standardized mortality ratio of 1.88 among patients with ADHD, although this was not statistically significant (95% CI, 0.83-4.26; *P* = .13). The Danish population-based cohort study by Dalsgaard et al^8^ described a 2-fold higher mortality rate in individuals with ADHD compared with those without it (mortality rate ratio, 2.07; 95% CI, 1.70-2.50), and London and Landes^9^ showed similar results, with an adjusted odds ratio of 1.78 (95% CI, 1.01-3.12). A 33-year follow up study^10,11^ of 207 boys with ADHD and 178 boys without ADHD and showed higher injury deaths among the boys with ADHD (10 of 207 vs 1 of 178; *P* = .01). However, shortcomings of previous studies have included insufficient number of deaths for cause-specific analysis,^7,8,9,10,11^ short follow-up periods,^9^ and limited capacity to adjust for important potential confounders such as comorbid psychiatric disorders.^7,9^

Understanding specific causes of mortality, including natural, unintentional injury, suicidal, or other injury causes of deaths, may provide insight for further definite interventions to reduce the risk of mortality in patients with ADHD. Hence, we designed this study to investigate and compare the risks of both overall and cause-specific mortality after controlling for possible confounders in people with or without ADHD who were identified using a large population-based data set.

## Methods

### Samples

**Figure 1.**
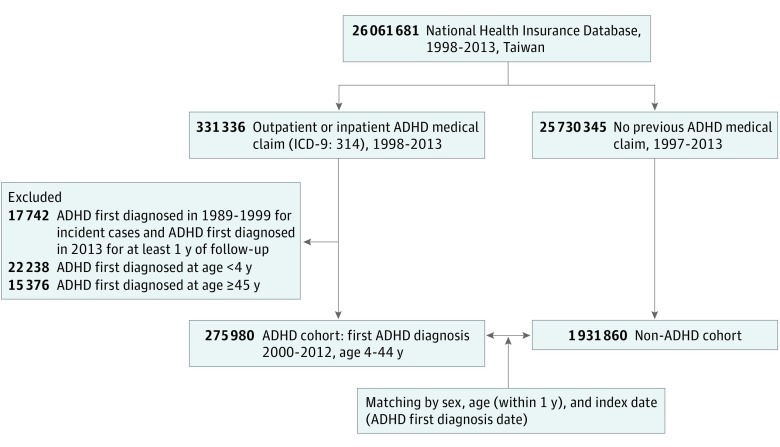
Flowchart of Data Collection in Study of Patients With Attention-Deficit/Hyperactivity Disorder (ADHD) and Matched Controls Without ADHD ICD-9 indicates *International Classification of Diseases, Ninth Revision*.

The government of Taiwan initiated a nationwide health insurance program—the National Health Insurance—on March 1, 1995. By December 2010, more than 23.07 million people were enrolled in the National Health Insurance system, yielding a coverage rate of 99.6% of residents in Taiwan. The National Health Research Institute then created the National Health Insurance Research Database from the National Health Insurance,^12^ deidentifying personal information and filing health care receipts. In this nationwide cohort study, we extracted information from the National Health Insurance Research Database on medical use among individuals aged 4 to 44 years for whom an ADHD diagnosis (*International Classification of Diseases, Ninth Revision* [*ICD-9*] code 314) was recorded between January 1, 2000, and December 31, 2012. The age range of 4 to 44 years was chosen because ADHD was unlikely to be diagnosed outside this range. The date of the first ADHD diagnosis was defined as the index date. The index date for participants in the comparison group was assigned as the date of the first ADHD diagnosis of their matched counterparts with ADHD. Sex- and age-matched comparison participants without a recorded diagnosis of ADHD were also selected at a ratio of 1:7 (Figure 1). The nationwide Mortality Register for 2000 to 2013 was provided by the Department of Health, the Executive Yuan of Taiwan.

The institutional review board of the National Taiwan Normal University approved this study. Written informed consent from study participants was not required because information from the National Health Insurance Research Database and the Mortality Register is anonymized and deidentified. This study followed the Strengthening the Reporting of Observational Studies in Epidemiology (STROBE) reporting guidelines.

### Study Variables

The main outcome measure for our cohorts was all-cause mortality. Deaths due to suicide, unintentional injury, homicide, and natural causes were also extracted as variables of interest. Because suicide mortality statistics are often underestimated and misclassified, suicide was defined as having *ICD-9* codes E950 to E959 (suicide) and E980 to E989 (injury undetermined whether accidentally or purposely inflicted). Mortality due to unintentional injuries was defined as *ICD-9* codes E800 to E949. Mortality by homicide was defined as *ICD-9* codes E960 to E969. Mortality by natural cause was defined as having causes other than suicide, unintentional injury, and homicide.

Demographic variables included age, sex, levels of income, and urbanization. The level of income served as an indicator of economic status and was classified into 3 categories of monthly income (given in New Taiwan dollars [NT$]): (1) less than NT$20 000, (2) NT$20 000 to NT$39 999, and (3) NT$40 000 and higher (US$1 = NT$32.1 in 2010). Other covariates included the presence at baseline of congenital anomaly (*ICD-9* codes 740-759), intellectual disability (*ICD-9* codes 317-319), depressive disorder (*ICD-9* codes 296.2, 296.3, 300.4, and 311), anxiety disorder (*ICD-9* codes 300.0, 300.01, 300.02, 300.2, 300.21, 300.23, and 300.3), autism (*ICD-9* code 299), substance use disorder (*ICD-9* codes, 303-304), and conduct disorder or oppositional defiant disorder (*ICD-9* codes 312 and 313.81) before the index date. Outpatient visits within the year before the index date, which served as an indicator of medical service use, was also a covariate and was classified into 3 categories: (1) 0 to 10, (2) 11 to 20, and (3) 21 times or more in a year.

### Statistical Analysis

The risk of total and sex-specific mortality during the follow-up period was calculated through survival analysis. The time function was calculated as the number of years from the index date to the date of death, withdrawal from the National Health Insurance program, or December 31, 2013 (the end of follow-up).

All-cause mortality was analyzed using a Cox regression model. Other causes of mortality during the follow-up period were considered as competing risk events for each of the specific-cause mortality outcomes (ie, suicide, unintentional injury, homicide, and natural causes). Modified log-rank test results were obtained using the Fine and Gray^13^ method, and adjusted cumulative incidences were calculated. Competing risk-adjusted Cox regression models^13,14^ were fitted to estimate the association of ADHD with specific-cause mortality after adjustment for covariates. Competing risk-adjusted hazard ratios (HRs) with 95% confidence intervals were calculated. Because the data fitted the proportional hazard assumption, Cox regression analysis was appropriate for the analysis. For multivariate analyses, model 1 incorporated adjustment for demographic variables and outpatient visits in the previous year, and model 2 incorporated further adjustments for baseline comorbidities of congenital anomaly, intellectual disability, depressive disorder, anxiety disorder, autism, substance use disorder, conduct disorder, or oppositional defiant disorder diagnosed before the index date. Secondary analyses were performed after stratification by sex.

Data management was performed using SAS statistical software version 9.4 (SAS Institute). Cumulative incidences and Cox model calculations in the competing risk analysis were performed using the package cmprsk in R statistical software version 3.5.3 (R Project for Statistical Computing).^14^ Two-tailed *P* < .05 (log-rank test) was considered statistically significant.

## Results

### Characteristics of ADHD and Control Groups

A total of 275 980 study participants with ADHD and 1 931 860 comparison participants without ADHD were identified. Table 1 depicts the demographic and clinical characteristics of the 2 groups. The mean (SD) age was 9.61 (5.74) years for both groups. Most of the participants were male (209 406 in the ADHD group; 1 465 842 in the non-ADHD group; 75.88% for both groups). Sex and age at index date were matched adequately. Comparing the 2 cohorts, those with ADHD had a higher level of urbanization; lower levels of income; higher proportions with congenital anomaly, intellectual disability, depressive disorder, autism, substance use disorder, conduct disorder, and oppositional defiant disorder; and higher numbers of outpatient visits. A total of 4321 participants from both cohorts died during the follow-up period (15.1 million person-years), including 727 in the ADHD group (0.26%) and 3594 in the non-ADHD group (0.19%). Of those who died, 75.1% (546) in the ADHD group and 79.4% (2852) in the non-ADHD group were male. The incidence rates of suicide, unintentional injuries, and homicide for the ADHD group were 0.62 per 10 000 person-years, 0.99 per 10 000 person-years, and 0.07 per 10 000 person-years, respectively. The incidence rates of suicide, unintentional injuries, and homicide for the non-ADHD group were 0.19 per 10 000 person-years, 0.82 per 10 000 person-years, and 0.04 per 10 000 person-years, respectively. The ADHD group had higher all-cause, suicide, unintentional injury, and homicide mortality than did the non-ADHD group.

**Table 1.** Demographic and Clinical Characteristics of Patients With ADHD and Sex- and Age-Matched Controls Without ADHD, Taiwan, 2000 to 2012^a^

Characteristic	No. (%)		*P* Value
	Group With ADHD	Group Without ADHD	
No.	275 980	1 931 860	
Sex			
Male	209 406 (75.88)	1 465 842 (75.88)	>.99
Female	66 574 (24.12)	466 018 (24.12)	
Age at index date, y			
4-11	222 967 (80.79)	1 560 769 (80.79)	>.99
12-17	38 266 (13.87)	267 862 (13.87)	
18-44	14 747 (5.34)	103 229 (5.34)	
Level of urbanization			
Rural	42 807 (15.51)	403 740 (20.90)	<.001
Urban	233 173 (84.49)	1 528 120 (79.10)	
Level of income, NT$			
<20 000^b^	84 743 (30.71)	578 681 (29.95)	<.001
20 000-39 999	132 499 (48.01)	965 387 (49.97)	
≥40 000	58 738 (21.28)	387 792 (20.07)	
Congenital anomaly			
Yes	4318 (1.56)	16 188 (0.84)	<.001
No	271 662 (98.44)	1 915 672 (99.16)	
Intellectual disability			
Yes	7417 (2.69)	5356 (0.28)	<.001
No	268 563 (97.31)	1 926 504 (99.72)	
Depressive disorder			
Yes	3335 (1.21)	1992 (0.10)	<.001
No	272 645 (98.79)	1 929 868 (99.9)	
Anxiety disorder			
Yes	271 490 (98.37)	1 927 564 (99.78)	<.001
No	4490 (1.63)	4296 (0.22)	
Autism			
Yes	5385 (1.95)	3277 (0.17)	<.001
No	270 595 (98.05)	1 928 583 (99.83)	
Substance use disorder			
Yes	2124 (0.77)	8834 (0.46)	<.001
No	273 856 (99.23)	1 923 026 (99.54)	
Conduct disorder or oppositional defiant disorder			
Yes	682 (0.25)	306 (0.02)	<.001
No	275 298 (99.75)	1 931 554 (99.98)	
Outpatient visits (times)^c^			
0-10	72 983 (26.45)	761 888 (39.44)	<.001
11-20	82 961 (30.06)	568 960 (29.45)	
≥21	120 036 (43.49)	601 012 (31.11)	

Abbreviation: ADHD, attention-deficit/hyperactivity disorder.

^a^ *International Classification of Diseases, Ninth Revision *codes include the following: congenital anomaly (740-759), intellectual disability (317-319), depressive disorder (296.2, 296.3, 300.4, and 311), anxiety disorders (300.0, 300.01, 300.02, 300.2, 300.21, 300.23, and 300.3), substance use disorder (303-304), conduct disorder or oppositional defiant disorder (312 and 313.81), suicide (950-959 and 980-989), unintentional injury (800-949), homicide (960-969), and natural-cause mortality (all-cause mortality excluded suicide, unintentional injury, and homicide).

^b^ 1 US$ = 32.1 NT$ (New Taiwan dollars) in 2010.

^c^ Past 1 year before the index date.

### Unadjusted (Univariate) Analysis

**Figure 2.**
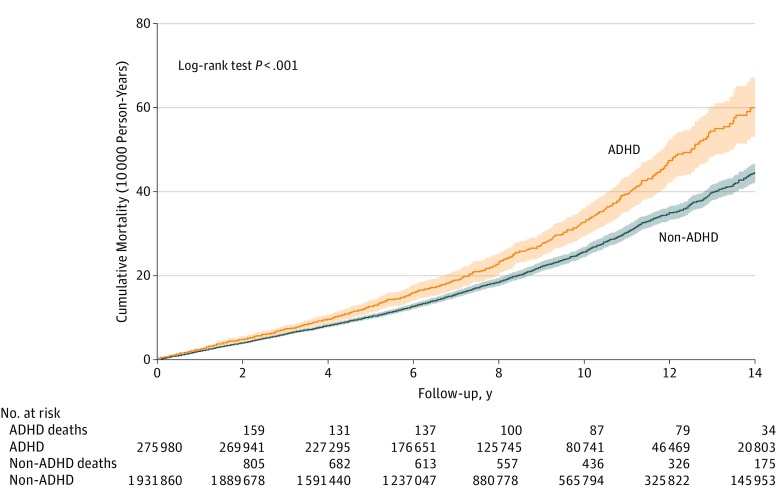
Comparison of All-Cause Cumulative Mortality Between Patients With Attention-Deficit/Hyperactivity Disorder (ADHD) and the Non-ADHD Group, Taiwan, 2000 to 2012 Shaded areas represent 95% CIs.

Table 2 shows unadjusted comparisons of total and sex-specific mortality between the ADHD and non-ADHD groups. The cumulative all-cause mortality rate in those with ADHD was significantly higher than in the non-ADHD comparison group (Figure 2). The crude mortality rate for the ADHD group was also significantly higher than that for the non-ADHD group (Table 2). The ADHD group had significantly higher all-cause mortality (HR, 1.42; 95% CI, 1.31-1.54; *P* < .001), suicide mortality (HR, 3.19; 95% CI, 2.56-3.97; *P* < .001), unintentional injury mortality (HR, 1.21; 95% CI, 1.03-1.41; *P* = .01), homicide mortality (HR, 2.04; 95% CI, 1.13-3.70; *P* = .007), and natural-cause mortality (HR, 1.30; 95% CI, 1.17-1.45; *P* < .001).

**Table 2.** Comparative Univariate Analysis of Mortality Between Patients With ADHD and a Group of Controls Without ADHD, Taiwan, 2000 to 2012

Outcome or Subgroup	Group With ADHD			Group Without ADHD			Estimate, HR (95% CI)^a^	*P *Value
	Deaths, No.	Person-Years, No.	Rate, Deaths/10 000 Person-Years	Deaths, No.	Person-Years, No.	Rate, Deaths/10 000 Person-Years		
All cause								
Total	727	189 2521	3.84	3594	13 254 591	2.71	1.42 (1.31-1.54)	<.001
Male	546	1 443 000	3.78	2852	10 105 209	2.82	1.34 (1.22-1.47)	<.001
Female	181	449 521	4.03	742	3 149 381	2.36	1.71 (1.45-2.01)	<.001
Suicide								
Total	117	1 892 521	0.62	257	13 254 591	0.19	3.19 (2.56-3.97)	<.001
Male	76	1 443 000	0.53	186	10 105 209	0.18	2.86 (2.19-3.74)	<.001
Female	41	449 521	0.91	71	3 149 381	0.23	4.04 (2.75-5.94)	<.001
Unintentional injury								
Total	188	1 892 521	0.99	1090	13 254 591	0.82	1.21 (1.03-1.41)	.01
Male	163	1 443 000	1.13	968	10 105 209	0.96	1.18 (1.01-1.39)	.03
Female	25	449 521	0.56	122	3 149 381	0.39	1.44 (0.93-2.21)	.15
Homicide								
Total	14	1 892 521	0.07	48	13 254 591	0.04	2.04 (1.13-3.70)	.007
Male	11	1 443 000	0.08	41	10 105 209	0.04	1.88 (1.01-3.65)	.02
Female	3	449 521	0.07	7	3 149 381	0.02	3.00 (0.78-11.61)	.25
Natural cause								
Total	408	1 892 521	2.17	2199	13 254 591	1.67	1.30 (1.17-1.45)	<.001
Male	295	1 443 000	2.07	1656	10 105 209	1.65	1.25 (1.11-1.42)	<.001
Female	113	449 521	2.51	543	3 149 381	1.72	1.46 (1.19-1.79)	<.001

Abbreviations: ADHD, attention-deficit/hyperactivity disorder; HR, hazard ratio.

^a^ All-cause mortality was analyzed by the log-rank test; specific-cause mortality was analyzed by the modified log-rank test using the Fine and Gray^13^ method.

### Adjusted (Multivariate) Analysis

Compared with the unadjusted value (HR, 1.42; 95% CI, 1.31-1.54), the strength of association between ADHD and all-cause mortality decreased slightly after model 1 adjustments (adjusted HR, 1.32; 95% CI, 1.22-1.43) and more substantially after model 2 adjustments (adjusted HR, 1.07; 95% CI, 1.00-1.17) were made. After adjustments for competing risks and covariates in model 2, having the diagnosis of ADHD remained associated with higher mortality from injury causes, including suicide (adjusted HR, 2.09; 95% CI, 1.62-2.71), unintentional injury (adjusted HR, 1.30; 95% CI, 1.10-1.52), and homicide (adjusted HR, 2.00; 95% CI, 1.09-3.68). However, no significant association was found between ADHD and mortality from natural causes after full adjustment (Table 3).

**Table 3.** Comparative Multivariate Analysis of Mortality Between Patients With Attention-Deficit/Hyperactivity Disorder and a Control Group Without Attention-Deficit/Hyperactivity Disorder, Taiwan, 2000 to 2012

Outcome or Subgroup	Adjusted Model 1^a^		Adjusted Model 2^b^	
	HR (95% CI)	*P* Value	HR (95% CI)	*P* Value
All-cause				
Total	1.32 (1.22-1.43)	<.001	1.07 (1.00-1.17)	.04
Male	1.26 (1.15-1.39)	<.001	1.04 (0.94-1.14)	.47
Female	1.52 (1.28-1.79)	<.001	1.24 (1.04-1.48)	.02
Suicide^c^				
Total	2.71 (2.16-3.40)	<.001	2.09 (1.62-2.71)	<.001
Male	2.48 (1.88-3.28)	<.001	2.06 (1.51-2.81)	<.001
Female	3.18 (2.13-4.75)	<.001	2.27 (1.44-3.59)	<.001
Unintentional injury^c^				
Total	1.32 (1.12-1.54)	.001	1.30 (1.10-1.52)	.002
Male	1.29 (1.09-1.53)	.003	1.28 (1.08-1.52)	.005
Female	1.44 (0.92-2.26)	.11	1.34 (0.83-2.18)	.23
Homicide^c^				
Total	2.10 (1.14-3.84)	.02	2.00 (1.09-3.68)	.03
Male	1.93 (0.98-3.79)	.06	1.80 (0.92-3.55)	.09
Female	3.06 (0.77-12.17	.11	3.06 (0.72-13.11)	.13
Natural cause^c^				
Total	1.14 (1.02-1.27)	.02	0.91 (0.80-1.15)	.18
Male	1.09 (0.96-1.24)	.17	0.83 (0.72-1.15)	.17
Female	1.28 (1.04-1.58)	.02	1.03 (0.82-1.30)	.81

Abbreviation: HR, hazard ratio.

^a^ Model 1: matched design by sex and age; adjusted by residence, level of income, and outpatient visits past 1 year before the index date.

^b^ Model 2: adjusted by model 1 and the presence of congenital anomaly (*International Classification of Diseases, Ninth Revision *codes 740-759), intellectual disability (codes 317-319), depressive disorder (codes 296.2, 296.3, 300.4, and 311), anxiety disorder (codes 300.0, 300.01, 300.02, 300.2, 300.21, 300.23, and 300.3), autism (code 299), substance use disorder (codes 303-304), and conduct disorder or oppositional defiant disorder (codes 312 and 313.81), which were diagnosed before the index date; and suicide (codes 950-959 and 980-989), unintentional injury (codes 800-949), homicide (codes 960-969), and natural-cause mortality (all-cause mortality excluded suicide, unintentional injury, and homicide).

^c^ Adjusted by other-cause mortality through competing risk-adjusted Cox regression.

## Discussion

In what we believe to be the largest sample evaluated to date, we investigated associations of ADHD with both all-cause and cause-specific mortality, incorporating adjustments for multiple potential confounding factors. Our overarching finding was that injury rather than natural-cause mortality was higher in individuals with ADHD than in a comparison cohort. Although unintentional injuries accounted for the highest number of deaths from injury causes, the highest excess risk of mortality in the ADHD group was for suicide.

Our finding of an association between ADHD and all-cause mortality is consistent with those reported by previous studies.^8,9^ However, another study by Barbaresi et al^7^ reported no increased risk of mortality among patients with ADHD, with standardized mortality ratios of 1.88 (95% CI, 0.83-4.26; *P* = .13).

Our finding for higher injury mortality was also similar to that reported by Dalsgaard et al.^8^ The longest follow-up study^10^ also showed higher rates of injury deaths among boys with ADHD than in a comparison group (10 of 207 vs 1 of 178; *P* = .01), but the low sample size cannot allow for further multiple analysis to control confounders or detect the separate effect of different types of injuries. On investigating cause-specific mortality further, we found that the ADHD group had higher suicide, unintentional injury, and homicide mortality rates than the comparison group. Previous studies have failed to identify a statistically significant risk of unintentional deaths in ADHD,^7,9^ although they have been limited by sample size. For example, the standardized mortality ratio was 1.70 (95% CI, 0.49-5.97) in the study by Barbaresi et al,^7^ with only 3 deaths due to unintentional injuries in their ADHD cohort. That study^7^ also showed significantly increased suicide deaths in patients with ADHD, but the authors were not able to adjust for confounding factors such as comorbidity. Some studies^8,9^ have shown that demographic characteristics (eg, age and sex) and psychiatric comorbidities, such as oppositional defiant disorder, conduct disorder, and substance use disorder, partially account for the risk of death in individuals with ADHD. In our study, after full adjustment for sociodemographic variables and comorbid psychiatric diagnoses, associations with natural-cause mortality were attenuated to near-null values, but increased risk for suicide or unintentional injury deaths held even when we controlled for comorbidities such as depression.

Underlying mechanisms associated with increased injury deaths in patients with ADHD, and the possible mitigating effects of treatment, warrant further investigation. Behaviors such as inattention, hyperactivity, and impulsivity may increase the risk of unintentional injuries,^15^ and unintentional injuries might result from an underestimation of the consequences of risk-taking behaviors. Previous studies have also described increased traffic unintentional injuries in children with ADHD and other mental disorders,^8,16,17^ and working memory impairment and impulsivity in ADHD could potentially be responsible.^18,19^ Medication use has been reported to have the potential to reduce such risks.^20^ On the other hand, poor quality of life, lack of social support, and emotional dysregulation in children with ADHD were shown and may also be associated with increased risk of suicidal behaviors.^21^

Potential sex differences were noted in our study, in that female patients with ADHD had significantly increased all-cause mortality risk in fully adjusted models, which was not present in male patients with ADHD. This finding is consistent with those of a Danish study^8^ that reported higher mortality risk in girls and women than in boys and men. Referral bias might be a reason because female patients with ADHD have been found to be referred for further evaluation less often than male patients.^22^ Therefore, women who receive a medical referral and receive a diagnosis of ADHD may present with much more severe behavioral symptoms and problems, thus accounting for greater mortality risk. In addition, girls with ADHD have been reported to receive less treatment with medication than boys, which may also contribute to their higher mortality risk.^8,23^ We found that men with ADHD were at a slightly increased risk of unintentional injury-related death compared with men without ADHD, a male predominance that was also previously reported for unintentional injury.^24,25^ However, other studies have not found sex differences in the risk of unintentional injury-related mortality in patients with ADHD.^20,26^

Although the risk of suicide-related mortality was significantly higher in patients with ADHD than in those without ADHD in our study, the absolute risk of mortality was low and suicide deaths were rare (n = 117), with natural-cause deaths (n = 408) and unintentional injury deaths (n = 188) accounting for a substantially higher number of deaths than suicide in the ADHD cohort. One previous study^8^ also reported unintentional injuries to be the most common cause of death in this patient group; however, of 107 deaths, information on cause was available for only 79 cases.

Findings from our study emphasize the importance of clinicians prioritizing the prevention of risk factors for injury-caused premature deaths in patients with ADHD, rather than compounding the stigma associated frequently with ADHD.^27^ Several studies have supported the evidence that ADHD medication can effectively reduce the risk of physical injury,^28^ fracture,^24^ traffic crashes,^20^ brain injury,^29,30^ and suicide.^31^ However, further research is still needed to estimate the potential effect of medication on injury mortality, including earlier identification and treatment initialization. Teaching emotional regulation, providing safety tips, educating on protective gear, and environmental modification may also be helpful.^21^

### Strengths and Limitations

To our knowledge, this is the first study using a national database to investigate associations between ADHD and different causes of mortality after controlling for associated comorbidities. The population-based cohort design diminished the likelihood of selection bias, beyond issues of access to diagnosis discussed later, and its prospective nature reduced the risk of reverse causality. We were also able to incorporate adjustment for a range of potential confounders. Notwithstanding, several limitations should be borne in mind when drawing inferences from our findings. First, the ADHD cohort was defined from routine health care data by medical diagnosis rather than by structured research-quality clinical interviews; furthermore, it is important to bear in mind that many patients with ADHD will not receive a clinical diagnosis and our findings cannot be assumed to generalize to those with undiagnosed disorder. Second, our study used observational data from the population in Taiwan, and its generalizability to other international settings cannot be assumed. Third, we were not able to consider and analyze the effects of ADHD medications or other potential contributing factors, such as family history and psychosocial stress.^32^ Some covariates adjusted for in our analyses would have provided incomplete estimation of underlying parameters of interest (eg, undiagnosed comorbidity not captured in health care records or occurring before the index date and limited measurements of socioeconomic status). For example, ADHD onset was measured by first recorded diagnosis, and the prediagnosis impact of ADHD has to be assumed; in addition, controlling for the baseline covariate of depression might be biasing downward the effect of ADHD if depression served as a mediator of ADHD to suicide. Future exploration of the possible effects of ADHD treatments and consideration of other confounders is required. Fourth, although the present study provided support that the population with ADHD is at risk for higher mortality due to injury causes, it did not reveal what might drive that risk. Further studies are warranted to explore the underlying mechanism.

## Conclusions

This study found that ADHD was associated with higher mortality, particularly increased risk of mortality from injury causes, compared with mortality in a control group without ADHD. The highest excess risk of mortality in the ADHD group was for suicide, even after adjusting for the comorbidities such as depression. The natural-cause mortality was attenuated to near-null values after adjustment for sociodemographic variables and comorbid psychiatric diagnoses. It is imperative to explore the underlying mechanisms of injury deaths in patients with ADHD to help develop effective prevention methods.
